# Blockchain Privacy and Scalability in a Decentralized Validated Energy Trading Context with Hyperledger Fabric

**DOI:** 10.3390/s22124585

**Published:** 2022-06-17

**Authors:** Johann Westphall, Jean Everson Martina

**Affiliations:** Computer Security Laboratory, Federal University of Santa Catarina (UFSC), Florianópolis 88040-370, Brazil; johann.westphall@posgrad.ufsc.br

**Keywords:** energy, blockchain, performance, scalability, privacy, hyperledger

## Abstract

Lower renewable energy generator prices are leading people to install solar panels to reduce their electricity bills or, in some cases, even sell the surplus generated energy to the grid and earn credits from the grid operator. Generally, they are limited to trading the energy they generate with the grid company, which has a dominant role in price determination. Decentralized energy markets might increase both market competitiveness and incentive to further people’s adoption of renewable energy, reducing security vulnerabilities and improving resiliency. Blockchain is a widely studied technology to provide decentralization for energy markets in this context. Scalability, privacy, market design, and user security are some of the open research topics. This work analyzes the literature related to blockchain and energy markets, proposes a model, implements it, performs experiments, and analyzes network scalability and data generation. The model, implemented with Hyperledger Fabric, enables validated clean energy trading with anonymized buyers to prevent consumption pattern exposure. The maximum transaction throughput was achieved with 5000 sensors, 5000 buyers, and 5000 sellers. The data generation rate by network and the baseline deployment costs were also analyzed to judge the network viability. Furthermore, this work provides empirical results on a topic that the literature lacks.

## 1. Introduction

Blockchain is a technology that enables a decentralized database in a Peer-to-Peer (P2P) context. It is widely known because of the Bitcoin cryptocurrency [1], and its structure is secure against tampering. Blockchain allows secure transactions between nodes without a TTP (Trusted Third-Party). It is considered to have the potential to enhance the role of consumers in the energy trading system by increasing security and reducing costs [2].

In most energy distribution systems, residences with renewable energy sources can only sell their excess produced energy to the utility company, which impedes broader price negotiation with multiple bidders. Researchers have explored blockchain as an enabler of a decentralized energy trading market, where residences could trade electricity with each other. These residence owners that buy and sell energy are called prosumers.

Beyond energy system improvement, the growth in renewable energy production brings environmental benefits. Considering the mentioned advantages and that blockchain is seen as a possible tool for achieving a decentralized energy market in a microgrid, research on the theme can lead to firmer conclusions on the viability of coupling blockchain with energy markets.

The authors of [3] surveyed blockchain-based energy projects. Even though they recognized the potential of utilizing blockchain to trade energy, they also found that the existing schemes were scarcely documented. The authors argued that future work must provide scientific depth to provide a convincing argument for utilizing blockchain in the energy system.

For [4], the blockchain enhances the energy internet’s different layers, but such a network requires privacy protections due to the risk of an attacker acquiring access to consumer behavior characteristics. The distributed ledger also strengthens the guarantee of the metrology devices and their certification, as the network can monitor changes to prevent tampering. Overall, the authors identify the demand for a multilevel approach for blockchains applied to the energy internet.

There are still open research topics on blockchain energy trading schemes in terms of system design, privacy methods to protect user data, and scalable solutions to deal with the amount of data collected through smart meters [2]. The authors of [5] argue that consumers might resist blockchain use in the energy markets due to a lack of privacy and that this context requires a blockchain with low latency and delay. These topics must be addressed to bring this type of system closer to adoption. We focus on analyzing the **scalability** of a blockchain energy trading model with **privacy**-preserving and energy source validation tools.

This work proposes a blockchain model for clean energy **validation** and **exchange**. Our model is implemented with Hyperledger Fabric, and it was designed based on Fabric’s capabilities. It uses a k-Times Anonymous Authentication (k-TAA) to ensure buyer anonymization, and it aims to perform efficient operations, given Fabric’s toolset. The model was not designed as an ultimate solution and can be further improved, centering on validating energy and privacy measures.

Our work’s main goal consists of determining the capabilities of blockchain use in energy markets, and we achieve that by means of our model design and experimental analysis. The experiments evaluated computational needs, storage needs, and transaction costs based on our model’s implementation. Our results indicate that our model would work in a **small neighborhood** on a performance basis with a reasonable cost per transaction.

### 1.1. Key Contributions

This work’s key contributions impact the three different areas of modeling, experimental analysis, and experiment setup. We introduce a model with clean energy validation, to augment the trust in the energy origin and attend to demands for clean energy by consumers. Furthermore, the need for consumer privacy is addressed in our model with buyer anonymization.

Our experimental analysis sheds light on the actual use of blockchain in energy markets by providing solid results. Thus, the applicability of blockchain to the field becomes more predictable regarding performance and cost. This addresses the problems found in the literature about lacking experimental results. Overall, future research might benefit from our experimental setup.

Lastly, we overcame technical challenges when implementing our model and performing experiments with Hyperledger Fabric. We implemented the Idemix support on the fabric-java-gateway to enable the buyers’ anonymization. Additionally, we ensured that the fabric-java-sdk could export the pseudonym used to sign the anonymous transactions for later determination of proof of ownership.

Modifications bypassing the *phantom read conflict* check on Hyperledger Fabric also represented a technical challenge and contribution. With our patch, chaincodes can define which functions can bypass such checks, expanding the applications supported by Hyperledger Fabric.

To reduce the measurement impact on the performance, we configured the chaincode container to save the maximum parallel requests supported by a peer. This ensures that the correct Golang channel amount will be allocated to process the average transaction delay.

### 1.2. Paper Structure

The rest of this work is structured as follows: Section 2 provides the theoretical background for our proposal, Section 3 presents a structured literature review, followed by Section 4, which elaborates on our blockchain energy trading model. In Section 5, we explain how the model was developed and deployed. Section 6 and Section 7 address the experiments’ design and the results analysis. Section 5 and Section 6 aim to help readers who desire to replicate our experiments, containing reasonable levels of detail. Finally, Section 8 provides conclusions and suggestions for future work.

## 2. Background

### 2.1. Blockchains

Blockchains are distributed ledgers, usually without a central authority, with a tamper-resistant and tamper-evident structure [6], enabled through public-key cryptography. This technology became well known for being part of the Bitcoin currency. A header and a data segment form each block. The transaction list is part of the data segment, while the block’s cryptographic hash, the previous block’s cryptographic hash reference, and a timestamp are parts of the header segment. A nonce is not always essential but depends on the consensus mechanism. Each transaction performed by a node is digitally signed and can be verified by all nodes using the public key.

There are two main categories of blockchain: permissionless and permissioned. Permissionless blockchains allow anyone to join the network, reading and writing to the ledger as desired. Permissionless blockchains are usually open source. Their consensus rewards publishing protocol-conforming blocks and requires some expense—work or stake—to validate blocks. These consensus constraints exist in permissionless blockchains due to the participation of unknown users who might act maliciously without possible accountability [6].

Permissioned blockchains have restrictions on who can take specific actions in the network. The level of restrictions can vary according to the permissioned blockchain policy. The ledger might be public for reading, but it may have access control for transacting. These settings depend on the context where the network is applied. With known users, the network consensus algorithm can be lighter than permissionless ledgers [6].

### 2.2. Hyperledger Fabric, a Permissioned Blockchain That Fits Energy Markets

In scenarios involving players with different roles and privileges, permissioned blockchains provide an extra security layer through access control [7]. Many permissioned blockchain tools differ on privacy level, and in terms of performance, programming language support, and architecture.

Hyperledger Fabric is an enterprise-grade permissioned blockchain that supports smart contracts in general-purpose programming languages [8]. All participants in the network are known, and they belong to an Organization which generates credentials through their CAs (Certificate Authorities).

Each Hyperledger Fabric transaction has to be endorsed by a set of peers responsible for storing ledgers and smart contracts. After that, the orderers receive the transactions and come to a consensus on their order, ensuring that all peers will store all transactions in the same sequence. When receiving the list of ordered transactions in a block, the peers judge the transactions’ validity. This transaction flow is called *execute–order–validate* [8].

Smart contracts can distinguish callers’ roles by reading their credential fields and, consequentially, applications can be developed with the assurance that the network will follow access control policies. At the same time, Hyperledger Fabric supports pseudo-anonymity through identity mixing algorithms using ZKP (Zero-Knowledge Proof), allowing participants to transact in the network with a certain degree of anonymity.

### 2.3. SmartData and Blockchains

SmartData is a standardized high-level Application Programming Interface (API) that facilitates IoT-related application development. It gathers a set of relevant attributes regarding data measured by sensors, including the unit, spatial location, timestamp, and reliability [9]. Blockchains receiving data from sensors may benefit from a standardized format, since it helps data interpretation. Smart contracts can be configured to act according to SmartData’s semantics.

Listing 1 demonstrates how SmartData is represented in JavaScript Object Notation (JSON) format. The field **version** determines if the device is stationary, in version “1.1”, or moving, version “1.2”. The metric is sensed by the device of identification **dev** and has a **value** related to a **unit**. An **uncertainty** degree about the data might be declared. The coordinates **x**, **y**, and **z** express the absolute spatial location associated with a specific instant represented by timestamp **t**. **Version** “1.2” also supports the SmartData cryptographic **signature** [10].

**Listing1.** Smart Data fields.{ “version”: unsigned char“unit”: unsigned long“value”: double“uncertainty”: unsigned long“x”: long“y”: long“z”: long“t”: unsigned long long“dev”: unsigned long“signature”: string }

## 3. Literature Review

The following questions drove this literature review:

- What are the current solutions presented in the literature about blockchain energy trading?
- What are the limitations of the current schemes?

We performed a search on IEEE (Institute of Electrical and Electronics Engineers) through the query in Listing 2. We selected the 18 most relevant papers to be analyzed through abstract and full-text reading. Two papers were immediately rejected. One of them was rejected due to having a publication date before 2018, and the other because its main focus was vehicular energy trading. We read the remaining 16 papers, plus the other eight relevant works cited by these 16, since their content complemented some aspects not covered by the ones found by query results. We assessed the full text of these 24 papers and selected those most related to our proposal.

**Listing2.** Research query.“All Metadata”:“Blockchain” AND “All Metadata”:“energy” AND (“All Metadata”: “trade” OR “All Metadata”:“trading”)

The authors of [11] propose a decentralized energy trading scheme enabled by an ESS (Energy Store System), with the price set by the Distribution System Operator (DSO). Sellers and buyers inform the energy amount to be sold or bought, and an Ethereum smart contract performs the matching. The work provides a general idea of a blockchain energy trading system. However, it is far from meeting the real world’s needs due to its simplicity and failure to mention scalability and privacy.

The work by [12] also proposes a blockchain energy trading solution using Ethereum. A contract is created and deployed on the blockchain for each energy transaction between consumer and prosumer. The consumer calls a function from the deployed contract offering a bid to the prosumer. When there is a match between bids, the energy is transferred, and the payment is performed. Even though the last cited work admits to being only a starting idea, it is interesting to mention its weaknesses. There is no market clearing process, and each transaction requires a new contract, which is unnecessary in terms of storage usage. The solution uses a consensus method like Proof-of-Work (PoW) in a private Ethereum network, whereas the authors could have taken advantage of the privacy and used a lighter consensus algorithm.

In [13], a blockchain-based energy trading scheme is designed with two layers. The first layer consists of a private blockchain to support local energy trading in a community context. In the second layer, regional energy aggregators trade energy cross-regionally. The energy aggregators are also responsible for coordinating the transactions locally, acting as third-party managers.

Payment off-chain and mixing private and consortium blockchains are considered solutions to protect user privacy. The work proposes a Proof-of-Stake consensus method based on each node’s credit score, and tokens signed by aggregators serve as energy credits to be consumed. The authors performed a mathematical analysis of their system performance and did not mention any blockchain tool. They suggest improving scalability and performance for future work.

The proposal in [14] describes a blockchain solution for energy trading between the DSO/Ancillary Services and their clients only. It does not enable P2P energy trading, while it recognizes that P2P trading’s complete implementation still faces challenges. The authors advocate using permissioned blockchain tools like Enterprise Ethereum and Hyperledger Fabric in energy schemes, as they allow more efficient consensus algorithms like Proof-of-Authority (PoA) and Proof-of-Stake (PoS). Lighter consensus methods are more suitable for constrained smart meters.

An Ethereum smart contract is presented. Each new block is added by authorized nodes using PoS consensus with grades calculated from past network behavior. The authors argue that blockchain can mitigate smart meters’ communication security vulnerabilities. As a future direction, the work suggests implementing a fully decentralized energy trading system.

The authors of [15] suggest a double-chained blockchain energy trading scheme. One chain stores smart contracts that report the power status of a user, and the other chain enables energy negotiation. The work argues that a decentralized blockchain energy trading scheme provides cyber resilience, eliminates monopoly, is transparent, and provides security. Research on optimizing the consensus and protecting the network against DoS (Denial of Service) is mentioned as future work. The paper describes only a generic blockchain energy trading scheme.

In [16], the authors propose a P2P energy trading scheme through an Ethereum private blockchain. On the scheme, the producer pays or requests for the network authorities to join the network. Once in the network, the producer deploys a smart contract to keep their price and available energy amount updated. The consumer performs a Commit to Pay transaction, putting the money on hold until the producer releases the energy. When energy is delivered, the consumer/buyer informs the network through an Energy Receipt Confirmation, and the funds are transferred to the producer. Transaction fees reward the block miners and serve as an incentive.

The authors present a proof-of-concept with two Raspberry Pi, using a Python extension to interact with the Ethereum blockchain and using Ether cryptocurrency as a payment method. They evaluated the system behavior with reliable and unreliable nodes. The authors also presented performance metrics like end-to-end delay, monetary cost, transaction throughput, and blockchain size.

The work of [17] presents a blockchain P2P energy trading scheme implemented with Hyperledger Fabric. It considers three main stakeholders: energy nodes, energy aggregators, and smart meters. Residents can trade with the utility (DSO) or with other residents. The architecture has its coin that can be converted to fiat money. The energy aggregator acts as a broker and manages the trades. Even though the scheme is not very detailed, the work describes and classifies different Hyperledger services. Fabric, Iroha, and Sawtooth are compared in consensus algorithm, consensus approach, and advantages.

The paper [18] brings a more detailed description and a more in-depth analysis of blockchain decentralized energy markets than the previously described papers. It shows a market structure closer to real centralized solutions like Nordpool [19]. The market is split into two phases: the day-ahead and the real-time market.

In their system, prosumers are classified as Type 1 or Type 2. Type 1 prosumers submit entirely to the power operator (DSO), and negotiations between them occur in the day-ahead market. Type 2 prosumers can trade with the DSO and between each other, but only in the real-time market. Since the authors’ predominant areas are electrical and electronic engineering, the scheme focuses on the Optimization Power Flow (OPF) solution and energy distribution.

They chose Hyperledger Fabric as an implementation tool because it met their requirements. The authors wanted a permissioned chain restricted to consumers/prosumers within the distribution area, efficient smart contract execution, practical consensus, and a model that protected users’ privacy. It is essential to highlight that they did not consider any other privacy protection mechanism beyond simply using Hyperledger Fabric. Even though they propose a decentralized market system, the DSO still plays a central authority role.

In [20], the blockchain energy trading model introduces side-chains (Plasma and Plasma Cash) on Ethereum to solve scalability problems and address smart meter computational constraints. Smart meters act as automated agents to trade energy. The energy trading process between microgrid’s participants happens on the side-chain, and the Merkle root of each block on the side chain is published in the main chain (Ethereum main net).

There is a centralized operator responsible for managing the side-chain. The authors cite higher throughput, reduced main net use, and the reliance on the main net as significant advantages for their model. They claim that future research should design real use cases of microgrid energy trading.

The authors of [21] focus on the blockchain energy trading system’s Internet of Things (IoT) component. They propose a proof-of-concept with Sigfox for smart meter communication and Ethereum as the blockchain tool. In their solution, smart meters send information and requests directly to the Sigfox Cloud, and blockchain miners are responsible for retrieving the data and publishing it on the chain.

The main contribution of [21] is to IoT communication. They tested the Sigfox technology communication range and concluded that, in a 1 km range, the Sigfox delivery success rate was 100%. The integration with the blockchain part of the system is mentioned as future work.

The Master thesis presented by [22] evaluates the feasibility of a blockchain energy trading system. It covers aspects like motivations for adopting such a system, (*Norwegian*) regulation, required infrastructure, challenges, desired blockchain characteristics, and implementation. The market design was divided into three parts: day-ahead, real-time, and load curtailment. The market clearing is performed off-chain by a node and verified on-chain.

The three market parts were simulated with Ethereum smart contracts, published on GitHub. The day-ahead and real-time markets were simulated with 600 nodes, while the load curtailment market was simulated with 25 nodes. The author analyzed the cost of the system based on the gas spent by all transactions.

Finally, the author classifies eight statements about the proposed scheme’s feasibility as true, false, or probable. The conclusion briefing was that the feasibility could not be proven, but some good evidence indicates that decentralized P2P blockchain energy trading is feasible. The work gives explicit and implicit future directions. Some are listed below.

- Perform tests on blockchain platforms other than Ethereum.
- Test the proposed system with real computers and smart meters.
- Propose privacy-preserving schemes for the blockchain energy market.
- Improve network scalability.

A privacy scheme with Multiparty Computation (MPC) is presented in [23]. Their algorithm is based on blockchain energy trading models, but the solution was implemented in C++ and never tested in a blockchain environment. The authors affirm that the simulation performed with actual energy data from Belgium indicates that the solution is feasible in a blockchain tool. The authors also present performance metrics regarding CPU operations, and their protocol was analyzed in terms of security aspects with the Universal Composability framework. Optimizing the MPC implementation is mentioned as future work.

The work [24] analyzes blockchain use in the energy sector regarding technology, economy, society, environment, and institutions in the Japanese context. The authors mention blockchain’s technological challenges when applied to energy markets. Throughput, latency, storage, and security are some of those challenges. Multi-chain communication, side chains, and off-chain storage are considered solutions for scalability problems.

A case pilot project in Misono, Japan, is presented with ten consumers, five prosumers, and one mall. The stakeholders exchange energy through a blockchain network and three possible power lines. They are equipped with solar panels, batteries, smart meters, and communications systems to interact with smart meters and the blockchain energy market. The chosen platform was Ethereum, with a PoA consensus.

The authors also affirm that privacy measures, such as pseudonymity, are critical next steps in the blockchain and energy integration context. Other research opportunities are enumerated: consensus mechanisms development, sharding, state channels, smart meter blockchain integration (via light clients), and privacy measures.

Our work proposes, implements, and validates an energy trading scheme in Hyperledger Fabric. We ensure the buyers’ privacy through identity mixing and analyze the implementation throughput and data generation rate to elucidate the proposal’s scalability. Buyers and sellers can exchange only validated energy generated in the past. The chaincode judges the energy generations as valid based on sensors measures periodically published to the chain. Our model considers the participation of utility companies and payment companies to settle the payment of anonymous buyers.

In Table 1, we compare the related work characteristics. Cells filled with an ✗ represent that the work has such characteristics. In the case of [22], the ✗ in the *Hyperledger* column indicates that Hyperledger was widely discussed, even though the solution was implemented using Ethereum.

The columns with a symbol like “
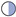
” indicate how deeply the work addressed such topics and the quality of their solution for each topic. The symbol “
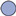
” represents maximum depth and quality, while “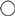 ” represents the lowest depth and quality. For example, [12] proposes a simple blockchain energy market model and does not cover some stakeholders, like the utility company. They do cover a context with only prosumers and consumers. However, the model of [22] supports three types of energy markets and considers the utility company. Therefore, in the **depth of market design** topic, [12] is rated “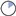 ” while [22] is rated “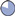 ”.

The work [24] was included based on the relevance of its experiments, and it did not come from the review process. Thus, we decided to add the work in this section to serve as a further base and comparison with our model.

## 4. Proposed Model

### 4.1. Model’s Literature Motivation

The work presented in [2] surveyed blockchain energy trading schemes and listed the main challenges of those systems. Among the challenges are low efficiency, privacy protection, and scalability issues. The authors claim that avoiding statistical predictions and behavior model analysis while preserving nodes’ rights and interests in the network may be a severe challenge.

In [3], the authors evaluate the whitepapers of blockchain-based peer-to-peer energy trading projects. They analyzed the projects’ characteristics, transaction elements, and energy ecosystem. The conclusion was that most of the evaluated projects were described too macroscopically and that future research must address this topic with more scientific depth.

Based on our literature review, Table 1 shows that there is still much potential for improvement in the blockchain energy trading schemes in terms of privacy, scalability, and market design. Our model addresses these three research gaps: *privacy*, *scalability*, and *market design*.

### 4.2. Entities and Their Actions

**Figure 1:**
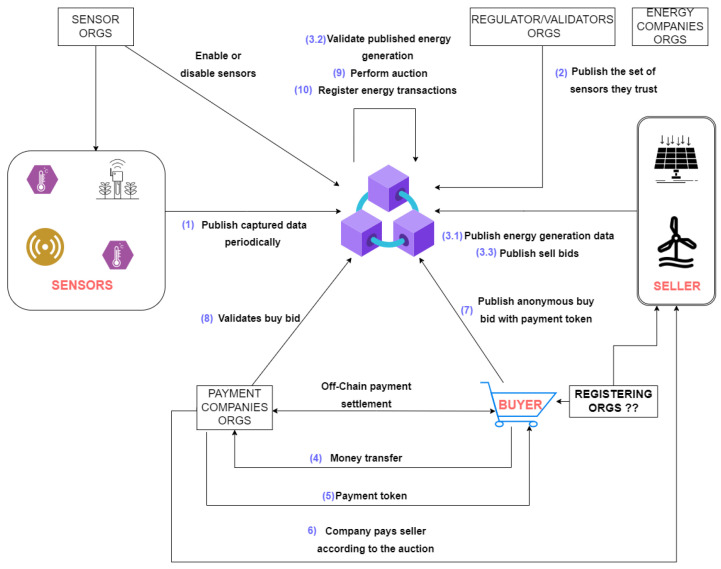
Model entities and their actions.

Our model consists of a blockchain network that aggregates five entities: **sensors**, **energy sellers**, **energy buyers**, **validators**, and **payment companies**. Each is entitled to perform their actions by calling different functions related to their roles on a **chaincode** (smart contract). Figure 1 presents the main actions of each network entity. Their actions will be described and explained in Section 4.2.1, Section 4.2.2, Section 4.2.3, Section 4.2.4 and Section 4.2.5, introducing their behavior in our model.

#### 4.2.1. Sensors

The sensors capture environment metrics and publish them to the blockchain network—action **1** in Figure 1. They can measure temperature, luminosity, humidity, wind speed, air pressure, electric current, and other relevant energy generation metrics. The sensors data is used to validate the energy sellers’ generation claims—action **3.2** in Figure 1. This process is described in further detail in Section 4.2.4.

Each sensor is registered to the network with its spatial coordinates, enabling it to infer an environment metric in a specific location in a specific time window. With data coming from many different sensors around a location, the network mitigates the attack of a sensor, intentionally or not, sending incorrect measurements to induce improper behavior.

#### 4.2.2. Energy Sellers

Energy sellers generate a specific energy type—solar, wind, hydroelectric, or other—and publish their energy generation in the blockchain by invoking a chaincode function—action **3.1** in Figure 1. They might be prosumers or local energy generation companies. The energy generation claims are validated before the energy amount is available for selling.

After the validation, a seller might publish sell bids so that buyers can match them—action **3.3** in Figure 1. The buyer’s payment company is responsible for paying the seller after the auction—actions **6** and **9** in Figure 1.

**Observation**: our model does not define the organization responsible for registering buyers and sellers. We assume that regulators or utility companies could do the registering role.

#### 4.2.3. Energy Buyers

Energy buyers use the network to buy a desired type of energy. For example, they might be concerned about pollution and want to buy only solar or wind energy. Since the network validates each energy bid, buyers have assurance about the origin of the energy they buy. The energy buyers prove to their energy distribution company that they bought energy in the network and receive discounts on electricity bills.

For example, if the buyer acquires 10 Kilowatt-hours (kWh) on the Energy Network and their meter registers the total consumption of 50 kWh, the utility company might charge only 40 kWh. This is possible because the utility company trusts the blockchain network to verify the energy generation since the buyer proves to have bought energy through the network. Therefore, when a buyer proves ownership of the transaction that bought energy, the company accepts it.

The buyer’s utility company knows that the buyer consumed 50 kWh based on their smart meter reads. However, when the buyer presents the acquirement proof of 10 kWh in the blockchain market to the utility company, it is only entitled to charge for the difference: 40 kWh.

Buyers publish an **anonymous** buy bid with a token from a payment company—action **7** in Figure 1. With this token, only the payment company **might** know the buyer’s identity while guaranteeing the seller that they will be paid.

#### 4.2.4. Validators and Validation

Every time an energy seller publishes their energy generation to the blockchain network, validators use the sensors’ data to judge whether the generation is legitimate. For example, a prosumer might have a solar panel that produces the maximum amount of 85 kWh on a sunny day. If this prosumer publishes an 85 kWh generation claim, but luminosity sensors near the prosumer indicate cloudy weather, the validators must reject the bid and not endorse it. Even if the prosumer’s smart meter indicates that they are feeding the grid 85 kWh, they might try to trick the energy network by selling dirty energy as clean energy.

State regulators, transmission line owners, private regulators, big energy sellers, or others can perform the validator role. Validators indicate to the network the sensors they trust—action **2** in Figure 1. When an energy seller publishes an energy generation claim, the nearby trusted sensors assist the claim validation—action **3.2** in Figure 1. A minimum number of validators are needed to endorse the energy generation claim.

**Figure 2:**
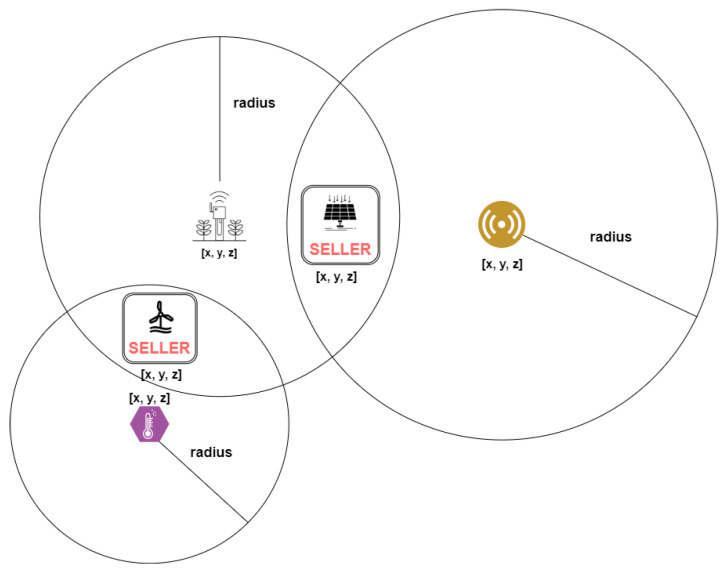
Considered physical topology.

Figure 2 shows how sensors are selected in terms of location. Sensors have spatial coordinates and a relevance radius that indicates the area where the sensor’s captured environment metric is equal or closely similar. The metric sensor unit must be related to the seller’s energy type. For example, to validate a **solar** energy generation claim, sensors that measure wind speed should not be selected, but luminosity sensors should.

Our model does not define the precise rules and criteria for validating the energy based on the environment metrics captured by sensors. It would require knowledge from the electrical engineering field. We only assume that such calculation is possible, and we represent it **symbolically** by averaging the sensors’ data near the seller and multiplying it to a constant.

#### 4.2.5. Payment Companies

Payment companies are organizations in the network responsible for settling off-chain payments between buyers and sellers. They receive funds from the buyers to send a token to compose the buy bid—actions **4** and **5** in Figure 1. This token represents a payment guarantee for the seller, who can withdraw the funds by presenting proof of transaction.

As soon as the buyer publishes their buy bid, they must request the validation of their bid by the payment company. After the request, the payment company validates the buy bid—action **8** in Figure 1—by informing the network how much funds the token covers in the buy bid. If a buyer tries to publish a buy bid offering more funds than the payment company claims to cover, the network will not allow the bid validation.

The validation avoids token theft and usage by a malicious user since there is no ownership information on the token. Without validation, a peer could read the token, reject the original buyer’s transaction, and utilize the token to buy energy for a third party.

Even though the token could be digitally signed by the payment company and reference the buyer credential to avoid the validation step, we opted not to add cryptography. A cryptographic token would create other problems, increasing the processing time due to cryptographic operations and decaying the model scalability. In these conditions, the token would need standardization across payment companies so that the chaincode could process it, unnecessarily increasing the chaincode’s complexity.

### 4.3. Actions Full Sequence

**Figure 3:**
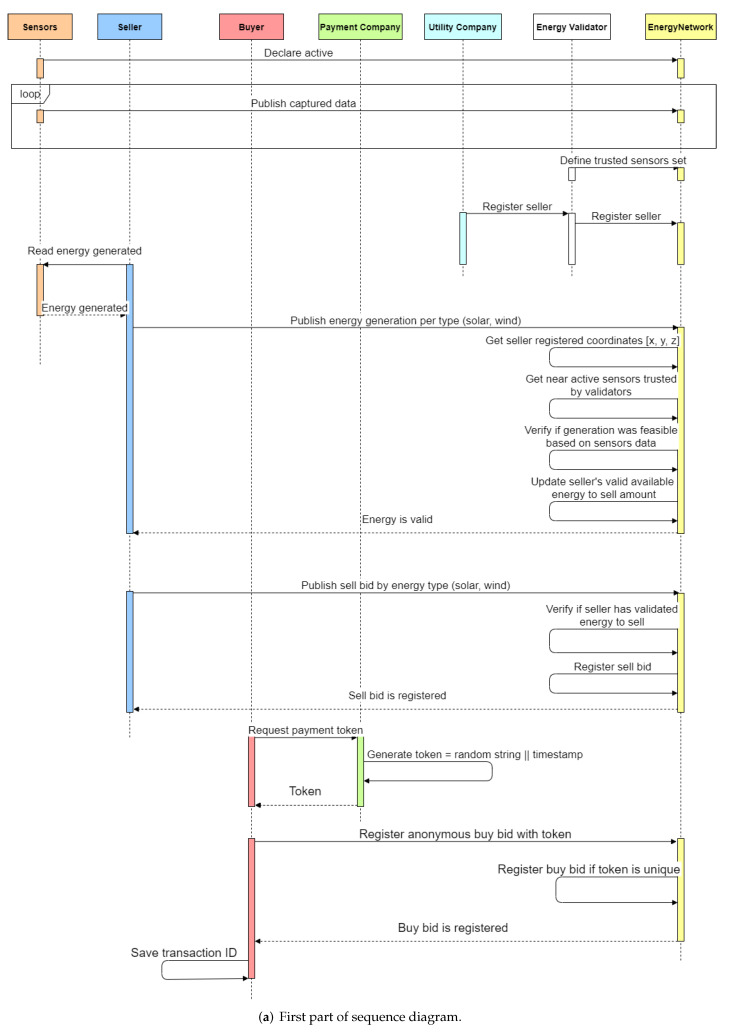

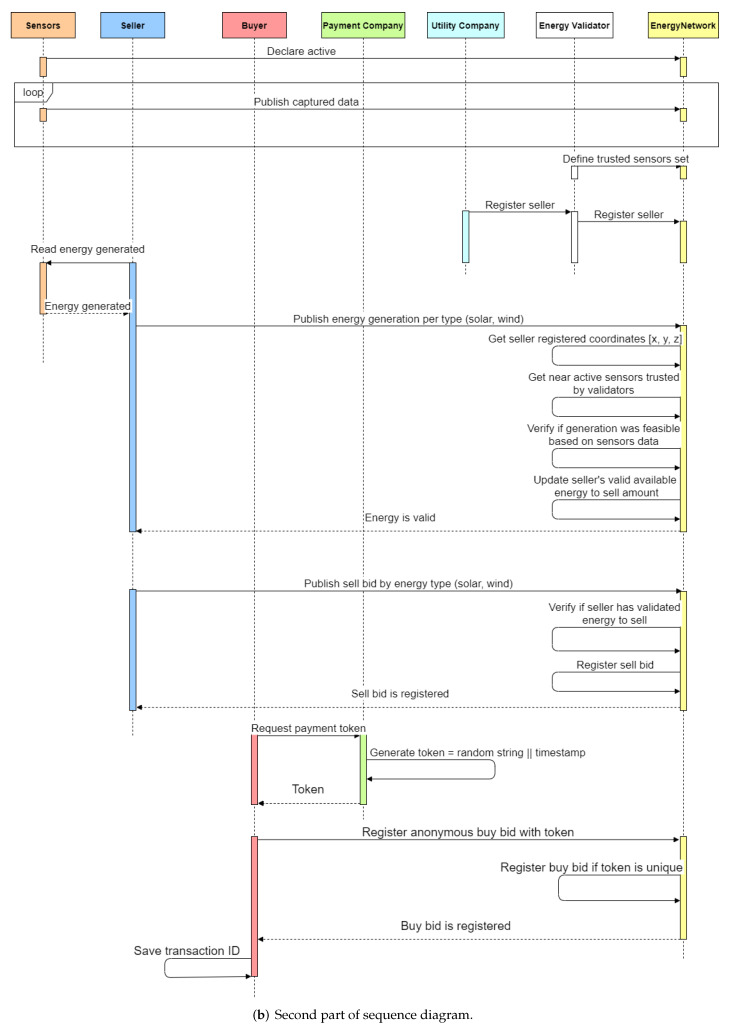
Sequence diagram.

The sequence diagram presented in Figure 3 shows a possible action sequence performed by entities. All these actions would happen in a real deployed network, simultaneously with multiple sellers, buyers’ payment companies, utility companies, and validators. However, the diagram clarifies the usual action sequence for each entity type.

First, each sensor declares itself active to the network and starts publishing its captured data. Following that, energy validators can define the sensor set they trust to be considered in their validation policy. As soon as the seller is registered, they or their automated gateway can publish energy generation claims. The chaincode judges the claims as valid or invalid based on the seller’s location and the sensors’ published data.

If the energy generated is ruled valid, the seller publishes a sell bid. A buyer desires to match this sell bid, and they request a token from the payment company before sending a buy bid. The buyer sends the buy bid to the network and requests the bid validation from the payment company, which validates it. After that, the buy bid participates in the network double auction and matches the sell bid.

The buy and sell bids are matched, and the energy transactions are registered to the ledger. Proving the bids’ issuance, buyers and sellers might request, respectively, energy bill discounts and payment for the sold energy. The utility company and the payment company respond accordingly after verifying the proofs.

The utility company tries to charge the energy customer for the consumption amount indicated on their smart meter. Nevertheless, if the consumer bought some energy on the blockchain network, they require a discount on their bill after providing the necessary evidence. In the seller’s case, they inform the payment token received after selling energy. The payment company verifies if the seller is the designated part of receiving the funds, and then the seller is paid.

### 4.4. Model’s Main Characteristics

The proposed model increases trust in the energy sellers because their energy generation claim is verified by multiple regulators, utility companies, or other validators based on the collected data of many sensors. Buyers can have significant assurance on the origin of the energy purchased. Every kWh exchanged through the network can be mapped to the sensors that have validated the generation.

Buyers can keep their anonymity while performing energy transactions to such an extent that network participants cannot infer the buyers’ energy consumption patterns. Even though our model does not specify if the energy bought is consumed instantly, it might be, depending on the deployment context. In such a scenario anonymizing the buyer becomes essential.

In our model, energy sold has to be generated in the **past** to simplify and avoid energy delivery verification complexity. Thus, sellers do not correspond to a Balance Responsible Party (BRP) and are not obliged to generate during a specific time window. This lack of responsibility might make it difficult for the utility companies to work to solve power imbalances. However, the vast amount of sensor data can serve as a counterbalance and, from another perspective, help to predict power imbalances.

Sell and buy bids can be partially matched, generating energy transactions registering the energy quantity and settlement price. With that, buyers prove their ownership of the bought energy and request discounts on their energy bills. Sellers contact the payment company to receive the funds related to their transactions.

### 4.5. Further Model Detail

Blockchains are heterogeneous and suit problems distinctly. Therefore, models’ specificities depend on the selected technology. The author of [25] considers proposals that explicitly define blockchain technology to be more grounded and realistic. Furthermore, they argue that building a hypothesis without defining the blockchain technology may hardly provide a concrete contribution.

For example, our model was designed based on the Hyperledger Fabric blockchain, which has an organization-oriented architecture. Thus, the same model would not fit Ethereum straightforwardly. For that reason, some further details of our model are elucidated in the implementation, available on our Git repository [26].

## 5. Model Development

### 5.1. Network Local Deployment

Hyperledger Fabric provides two alternatives for network deployment and testing: the testnet and production networks. The testnet is designed to run locally in a pre-defined network structure, with a couple of peers and a single orderer. It provides a simple and easily deployable environment so that application designers can execute tests without deploying a production network, which is more complex.

The production network is the one used network in real environments. It allows the creation of as many peers, orderers, admins, and clients as defined. All of our tests are performed in a production network so that the results are closer to real applications.

We developed a bash script and some python scripts for easily creating different networks. With these tools, we could deploy a network with as many organizations as necessary. Each organization can have as many admins, clients, orderers, and peers as wished. The script code is available on GitHub [26].

### 5.2. Chaincode Deployment

This section presents the developed chaincode that executes in every network peer. It contains functions to execute the actions displayed by the sequence diagram in Figure 3.

Go was the chosen language to implement our model since this is a general recommendation for developers because it matches the Hyperledger Fabric implementation language. Usually, the new chaincode features become available first in Go, besides generating smaller docker images. Also, the authors of [27] analyzed each chaincode language—Go, Java, and JavaScript—and identified a better performance on contracts written in Go.

Hyperledger Fabric transactions interact with the ledger and World State, usually reading or writing to a State. Fabric’s State database stores *key-value* pairs representing different states, with the *key* as a string and the *value* stored as bytes. In our chaincode, we store Go structs after serializing them to Protocol Buffers (protobuf). These structs represent data required by our model like sensor data, buy bids, sell bids, energy generated by sellers, and energy transactions.

A key can be **simple** with a single name identification with only *utf-8* characters. Another possibility is the **composite** one, when the goal is to form the key with an *object type* and many attributes. The *object type* and the attributes are placed in sequence and separated by the minimum Unicode character (∖u0000), aiming to avoid collisions with **simple** keys.

Hyperledger Fabric enables fetching State *values* by providing the whole key or a key prefix. Deletions and insertions are also possible by providing the full State key. We designed our chaincode to ensure efficient data queries given Fabric’s database structure.

Hyperledger Fabric supports two databases to store the ledger and World State. Go LevelDB has a simple key-value architecture and only supports key, key range, and composite key queries [28]. On the other hand, CouchDB supports more diverse queries, as long as the data is modeled in JSON format. After some tests presented and discussed in Section 7.1, we opted for **LevelDB** due to significantly better performance results.

The whole chaincode implementation is available at [26], and it may elucidate specific processes like energy validation and double auction. Furthermore, some small but significant modifications to Hyperledger Fabric’s source code were required to support our chaincode execution and experiments. One of them was the removal of *phantom read conflict* check.

### 5.3. Application Deployment

After the network is deployed, applications can interact with it through chaincodes. Hyperledger Fabric provides Software Development Kits (SDKs) and gateways to develop these applications. SDKs are available in Java, Python, Go, and JavaScript. We opted for the Java SDK because it was the only one with almost full Idemix support, enabling the anonymization of the buyers’ transactions. In our case, we implemented one application for each stakeholder in our network: buyer, seller, sensor, utility company, and payment company.

The sensor application generates random data concerning the environment and publishes it to the ledger. Buyers publish anonymous transactions, enabled by Idemix, with a payment token generated and validated by the payment company. Sellers publish energy generation claims, which are validated by the network. After the validation, they can sell the desired amount of the validated energy. The payment and the utility company were programmed as Hypertext Transfer Protocol (HTTP) servers that receive requests from buyers or sellers and interact with the ledger to grant payments or energy bill discounts.

Some modifications to the SDK and Gateway were required to satisfy our model and experiment needs. One of them regards the implementation of Idemix support on Fabric Gateway for Java [29]. Another relevant modification was on the Fabric SDK for Java [30] to enable storing Idemix credentials for later ownership proving. The complete changes are available on our repository [26].

### 5.4. Network AWS Deployment

Experiments in a local context are difficult to scale and are not always representative of a real environment. For this reason, we created the necessary scripts and architecture to adapt the local deployment to an AWS one. AWS offers vast cloud solutions with products and services related to many different knowledge areas, from databases to robotics. It counts on hosts located in the world’s main regions and allows customers to choose where applications are deployed. The users have a variety of machine configurations at their disposal, depending on resource requirements and budget.

To deploy our cloud blockchain network, we selected the **Elastic Compute Cloud (EC2)**, a configurable virtual computing environment. The general steps to set up a network go through selecting an operating system image, or Amazon Machine Image (AMI), choosing the hardware configurations for each virtual machine (instance) and defining their storage type (SSD, IOPS SSD, hard disk).

Powerful compute instances were required to run our experiments as close as possible to a real blockchain network for energy transactions—with many sellers, buyers, and sensors. We analyzed the costs to run instances with at least 32 cores and realized the cost disparity between arm and intel instances for budgeting the experiments. The on-demand Linux pricing for the type *m5.16xlarge* (intel) was 3.232 USD per hour, while the arm equivalent *m6g.16xlarge* cost 1.6191 USD per hour.

This 50% difference motivated us to deploy our experiments fully on arm instances. Furthermore, AWS claims the arm instances to be 60% more energy efficient than the Intel ones, which better fits the clean energy context of this work. Changes on the Fabric SDKs and the peer docker images were required to enable the execution of our experiments on arm architecture. All these changes are presented in our GitHub repository [26].

#### EnergyNetwork Deploy Steps in AWS

The first step involved creating modified *AMIs* from the quick start *Ubuntu Server 20.04 LTS (HVM)* image (ami-0a6638920f7143fb2) for arm architecture. We started an instance with this quick start image and installed all required packages, including our patched Fabric docker images, the *fabric-sdk-java*, and the *fabric-gateway-java*. The script “*create-ami-arm.sh*” in our GitHub repository [26] was executed to generate our custom Ubuntu AMI.

**Figure 4:**
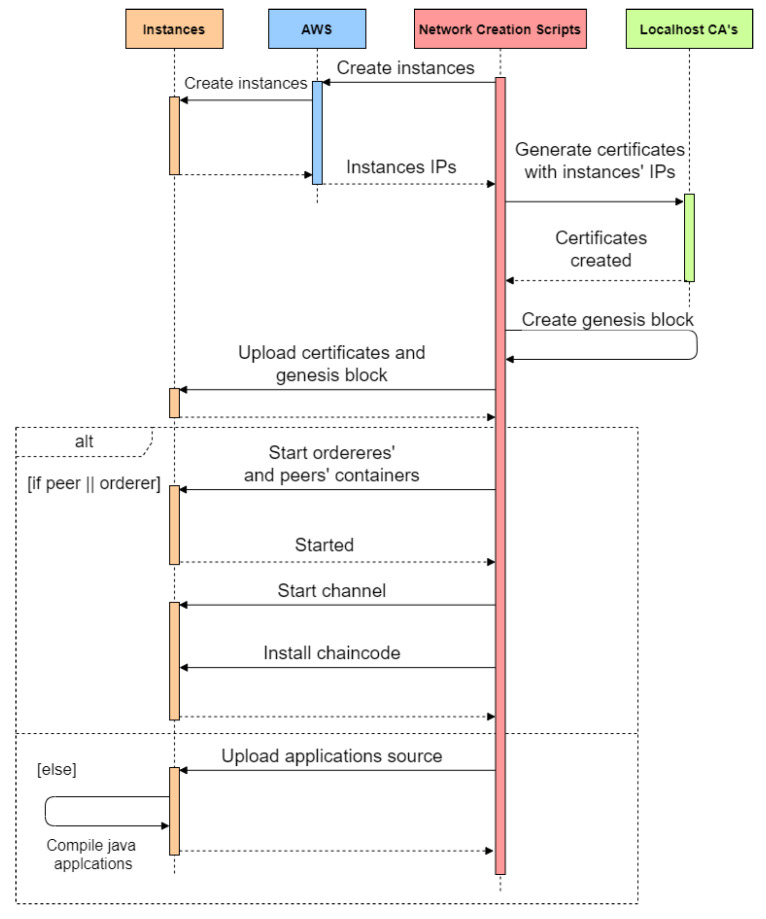
AWS deploy high-level sequence.

Next, we adapted the local docker deploy script to the “*automated-aws-creatio- n.sh*” script, which receives the instance types as arguments, reads the configuration files, and deploys the network in AWS. Figure 4 shows the high-level operations of the deployment with peers, orderers, a chaincode, and application instances. Localhost Certificate Authorities (CAs) generate the certificates for the hostnames or IP addresses allocated by AWS to each instance.

## 6. Experiment Design

### 6.1. Experiment Design Goals

We designed our experiments to obtain answers to our research questions about the scalability of blockchain energy trading with a guarantee of origin. The focus is on how a Hyperledger Fabric network running a chaincode to validate and transact energy handles vast amounts of transactions. For that, we test different network configurations, varying the quantities of buyers, sellers, and sensors.

The frequency of transactions—energy validation, auction, buying, and selling—is also configurable. With these settings, we can discover how a specific configuration change impacts the network’s performance. For example, increasing the sensors’ data publication frequency might require more peers to keep the network operational.

### 6.2. Experiment Adaptations

Our applications and chaincode functions had to be adapted to support larger-scale experiments. HTTP servers and creating a certificate for each buyer, seller, and sensor had to be eliminated. Chaincode execution averages had to be calculated without affecting performance.

The applications were adapted to simulate multiple buyers, sellers, and sensors. Each one has a specific purpose of simulating only one entity type—buyer, seller, or sensor. These applications receive arguments about the transaction period, the number of transactions, path to certificates, or idemix credentials. Each application interacts with the blockchain through a single Java gateway.

During the experiments, the payment and utility companies’ HTTP servers were eliminated to prevent them from becoming the throughput bottleneck and interfering with the blockchain measuring process. For taking chaincode execution time metrics, we ensured coordination and prevented undesired blocking of the chaincode’s functions with the help of the Go channel communication.

### 6.3. Experiment Rounds and Its Configurations

We structured our scripts to execute experiments in rounds, defined by a set of configurations and results. The configurations encompass blockchain configurations, like the number of orderers, and application configurations, like the sensors’ quantity. Results describe metrics and statistics within a single experiment round.

Each experiment round has its own set of configurations regarding the blockchain—peers, orderers, organizations—and the applications—buyers, sellers, sensors, and transaction frequency. The following configurations are determined before network creation:

1. Organizations
2. Number of peers
3. Number of orderers
4. Number of application instances
5. Peer concurrency limits
6. Peers AWS instance type
7. Orderers AWS instance type
8. Applications AWS instance type
9. Batch size
10. Batch timeout

After the network creation, multiple experiments might be performed, but the only possible configuration changes are related to the applications, displayed in Listing 3. Experiments with different blockchain configurations require complete network recreation.

**Listing3.** Application test configuration.#quantity per cli-applicationsensors:quantity: 1000unit: 3834792229#Interval in mspublishinterval: 5000publishquantity: 20msp: UFSCsellers:quantity: 1000#Interval in mspublishinterval: 5000publishquantity: 5msp: UFSCbuyers:quantity: 1000#Interval in mspublishinterval: 5000publishquantity: 10msp: IDEMIXORG#Interval in msauctioninterval: 30,000

#### Experiment Round Results

During an experiment round, the peers’, chaincodes’, orderers’, and applications’ metrics are continuously fetched through SSH and dumped into files. The “docker stats” command is executed in the background until all applications finish their transactions. These CPU, memory, network, and disk stats are later processed by a python script that generates plots from the data.

We periodically get the average execution time of each chaincode function. The initial and final file system sizes of peers and orderers are also measured to identify how much data an experiment round generated. In the end, we retrieve the peers’, orderers’, and applications’ logs to verify if any abnormal behavior happened. Eventually, when stress testing the network, some transactions might be rejected, or connection timeouts might appear in the logging files.

### 6.4. Experiments with Different AWS Instances

We evaluated the AWS instances’ performance in a context with **one orderer**, **one peer**, and **one or two application instances**. Algorithm 1 provides a high-level idea about how we performed these experiment tests. We started with the limited arm instance *t4g.micro* for the peer, orderer, and application. The test load was constantly increased by growing the number of sellers, buyers, and sensors until the log indicated failures.

When the logs presented failures, we interpreted the test result to find what instance—peer, orderer, or application—needed an upgrade to support the test load or if some Hyperledger Fabric’s configuration should be changed. After the instance upgrade or configuration change, the experiment round was rerun with the same load and was expected to work.
**Algorithm 1** Experiment tests.1:**procedure**TestInstancesLimit2:    peerInstance←“t4g.micro”3:    ordererInstance←“t4g.micro”4:    applicationsInstance←“t4g.micro”5:    testConfiguration← getSmallLoadConfiguration6:    **while** weWantToPerformAnotherRound **do**7:        logs←runExperimentRound(testConfiguration)8:        **if** logsPresentsErrors(logs) **then**9:           configurationNeedsChange←identifyConfigChangeNeed(logs)10:           **if** configurationNeedsChange **then**11:               testConfiguration←changeSomeFabricConfig(testConfiguration)12:               **continue**13:           **end if**14:           entityToBeUpgraded←identifyWhoNeedsUpgrade(logs)15:           **if** entityToBeUpgraded=“peer” **then**16:               peerInstance←upgradeInstance(peerInstance)17:           **else if** entityToBeUpgraded=“orderer” **then**18:               ordererInstance←upgradeInstance(ordererInstance)19:           **else**20:               applicationsInstance←upgradeInstance(applicationsInstance)21:           **end if**22:        **else**23:           testConfiguration←increaseLoad(testConfiguration)24:        **end if**25:    **end while**26:**end procedure**

#### 6.4.1. Phase 1 Experiment

The first phase of the experiments ran with all sensors participating in all energy validations. It raised our awareness about limiting the number of sensors after analyzing the steep average time increase in the execution of the energy validation function. This phase’s results are discussed in Section 7.2.1.

In Phase 1, the transaction publication quantities and intervals of each entity type were equivalent to those displayed in Listing 3. Nevertheless, the numbers of sellers, sensors, and buyers were varied. This phase ended with a particular failure regarding the **peer endorsing concurrency limit**, set by default to 2500 concurrent endorsing requests.

#### 6.4.2. Phase 2 Experiment

In the Phase 2 experiments, we increased the peer’s concurrency limit and avoided this failure, provided that the peer instance has the required computing power. The limit was set to 1 million concurrent requests to practically eliminate any concurrency restrictions, even though we never experimented with 1 million concurrent transactions. As in Phase 1, besides the number of buyers, sellers, and sensors, the Phase 2 configuration was the same as presented by Listing 3.

We also increased the chaincode container RAM allocation since the chaincode started to respond to more simultaneous requests after the concurrency limit increase. The number of sensors participating in each energy generation validation was limited to **5** to represent a more realistic scenario. This phase’s results are presented in Section 7.2.2.

#### 6.4.3. Phase 3 Experiment

The authors of [31] recommend a larger block size in Hyperledger Fabric environments with high throughput and lower latency demands. Considering that, in Phase 3, we performed tests with different block sizes and block timeout values. These configurations are set in the “configtx.yaml” file of the deployed network. This file in our repository [26] presents the parameters that deal with block configurations, containing brief documentation explaining each field. The results of this phase are presented and discussed in Section 7.2.3.

### 6.5. Data Generation Rate Experiments

Sensors continuously sending SmartData to the blockchain coupled with energy bid submissions might generate a massive amount of data, and knowing this data generation rate can point to network limitations. For that reason, we evaluated the data quantity generated in Phases 2 and 3—with sensors, buyers, and sellers. The peer’s and orderer’s root (“/”) file systems’ sizes were measured before and after the experiment rounds. We intended to draw conclusions discussing whether the data rate could be considered a problem with the results.

## 7. Results

### 7.1. Preliminary Metrics—CouchDB vs. Go LevelDB

The preliminary metrics provided base metrics to decide on the best performance chaincode design. We compared the time to retrieve a set of ledger states between CouchDB’s and LevelDB’s queries since Hyperledger Fabric supports both. The time measures were taken in a single machine with two orderers, two peers, and two CouchDBs for each peer, all entities running in docker containers. The preliminary metrics’ intent is not to estimate the World State’s access time in a real deployment context but to find the differences between the two databases, which would likely be proportional in a real deployment.

Our chaincode final version serializes the structs using Remote Procedure Calls (gRPC) to take advantage of more efficient storage. However, in these preliminary experiments, the chaincode serialized structs to **JSON** format, which is required for using CouchDB with Hyperledger Fabric. All structs are available at our repository [26].

In our chaincode’s context, we measured some preliminary database query times to decide on the most appropriate database. Our initial metrics involved querying *SmartData* in a determined timestamp range from the World State. The other two queries regarded retrieving *SellerInfo* by its *SmartMeter* ID and fetching *SellBids*/*BuyBids* to perform the double auction. LevelDB was considered more appropriate for our chaincode than CouchDB based on the metrics.

At first sight, it might seem obvious that a CouchDB running as an extra docker container, communicating with the peer using the network, and performing JSON queries would be slower than a LevelDB peer-local with only *Key-Value* queries. However, we considered that JSON queries could filter more data in the database, optimize network usage, and avoid data filtering on the chaincode. Also, we created *indexes* for the JSON queries, as recommended in Hyperledger Fabric’s documentation, for optimization [28].

Since we performed these preliminary experiments on a single machine, the network delay effect was almost irrelevant to the communications between peers and their CouchDB instance and between peers and their chaincode instance. LevelDB was **1000 times** faster at fetching *SmartData*, and **six times** faster at fetching *SellerInfo*, leading to an auction **ten times** faster than CouchDB.

### 7.2. Experiment Results

This section presents and discusses our main 3-phase experiments with our proposal’s implementation. We analyze the transaction throughput, data generation rate, and estimated deployment costs with the experiment metrics. Then, the model’s viability is addressed, followed by a comparison with the related work solutions.

#### 7.2.1. Phase 1 Experiment Results

The first phase of the experiments ran with all sensors participating in all energy validations. It raised our awareness about limiting the number of sensors. Some configurations and results of the first phase are presented in Table 2. Each row represents a configuration that failed in an experiment round. The first column contains which entity—application, orderer, or peer—indicated failure, with the AWS instance name highlighted in red. An instance upgrade happened after every failure, usually related to not properly supporting the experiment processing demands.

The rightmost columns of Table 2 present the numbers of sellers, sensors, and buyers for each round that presented a failure. We had to upgrade the instances to continuously increase the network participant capacity and go from 600 to 3000 sellers, sensors, and buyers.

Table 2’s last line shows a very specific failure regarding the **peer endorsing concurrency limit**, which is set by default to 2500 concurrent endorsing requests. In the Phase 2 experiments described in Section 7.2.2, we increase this limit and avoid such failure, provided that the peer instance has the required computing power.

#### 7.2.2. Phase 2 Experiment Results

Table 3 presents the results of the Phase 2 experiment. We could scale the network capacity from 2000 sellers, 2000 sensors, and 2000 buyers, in the first round, to 3500 sellers, 3500 sensors, and 3500 buyers in the last one. The orderer had to be upgraded up to *c6g.4xlarge* AWS instance, with 8 cores, 32 Gibibyte (GiB), and 10 Gigabits per second (Gbps) network capacity. The peer had to be scaled to the *c6g.8xlarge* instance, with 16 cores, 64 GiB of RAM, and up to 25 Gbps network.

Only **five** sensors were selected to validate energy generation claims in this phase, different from Phase 1. This was probably the main reason for the increase in the network participant capacity. In round 4, the chaincode had a memory limit failure, and its container’s memory was upgraded to 4 GiB. Later, we even allocated 12 GiB to the chaincode to prevent this failure in the following experiment rounds.

The last three experiment rounds demonstrate the difficulty of increasing sellers, sensors, and buyers. Despite three consecutive upgrades, the logs always presented some type of failure indication regarding network capacity. These results made us end Phase 2 and start Phase 3.

#### 7.2.3. Phase 3 Experiment Results

The two previous experiment phases had the default maximum block interval of **2 s**, with at most **500** transactions in a block or the maximum block preferred size of **2 MB**. The last three lines of Table 3 indicate difficulties in scaling beyond 3500 sellers, 3500 sensors, and 3500 buyers. We tested different block intervals, maximum transaction numbers, and maximum preferred block sizes in this phase, expecting to increase the scalability limits achieved in Phase 2.

We considered the average transaction size of 4 Kilobyte (KB) to keep always a *Block preferred size* with the capacity to fit *Block transaction limit* transactions. Otherwise, the blocks’ creation would be triggered by surpassing the *Block preferred size* and would never group *Block transaction limit* transactions.

Round 1 of this phase, in Table 4, presented failures in the application’s instance, indicating resource exhaustion. Thus, after round 1, we added a second application instance to divide the sensors, sellers, and buyers simulation load. For example, instead of making a single application instance simulate 3500 buyers, two instances simulate 1750 buyers each.

Unlike the previous phases, we decided to include the successful experiment rounds to explicit the network configurations that worked adequately in this one. In round 2, all logs indicated a successful execution when dealing with 3500 sensors, 3500 sellers, and 3500 buyers simultaneously.

In the third round, we tried to increase the quantity of each entity type to 5000 and failed, but round 4 made clear that the *Block transaction limit* should be increased from 2000 to 10,000 maximum transactions in a single block to run without errors. After performing tests with 6000 sellers, 6000 sensors, and 6000 buyers in round 5, the chaincode container ran out of memory, and we allocated 12 GiB for the following rounds.

Rounds 6 through 8 failed due to excessive reporting of orderer timeout in the applications’ logs, which was **60 s**. This means that after sending transactions to the orderer, the applications repeatedly did not receive a response after 60 s. Thus, the Hyperledger Fabric network could not absorb such high throughput with the configurations displayed in Table 4.

In round 7, we upgraded the orderer instance to a *c6g.16xlarge* AWS instance and increased the *Block transaction limit* to 20,000. After it failed, our final experiment round had a 20-s *Block interval* and 30,000 maximum messages per block. However, the final round also presented excessive orderer timeouts and was classified as a failure.

It is important to emphasize that if the applications’ orderer timeout limit were set to a value greater than **60 s**, rounds like the 7th would probably run without error indications. Still, that was **our** criteria for an acceptable orderer timeout, even though we recognize that it depends on the application scenario.

**Observation**: We identified that the peer auction events were not sent to the applications in this phase. This prevented the auction transactions from being called and sell bids being matched to buy bids. However, the auction transactions represent an irrelevant share of all transactions in a throughput perspective—lower than 0.02%. The cause of such failure was not identified. It could have been caused by the higher peer demand or the larger batch interval. Thus, regardless of this small failure, the orderer could handle the transaction throughput indicated as “success” by Table 4.

### 7.3. Data Generation Rate

Every experiment round in Phases 1, 2, and 3 measured the orderer’s and peer’s file system size to identify the proportion of data generation rate. The file systems were measured at two distinct moments. First, before any application issues any transaction to the network, at the round beginning. Second, after all the applications published all their transactions.

Table 5 and Table 6 present, respectively, the orderer’s and peer’s file system sizes corresponding to the experiment rounds performed in Phase 3. The file systems’ sizes presented in Table 5 and Table 6 are proportional to the number of sellers, sensors, and buyers. Table 7 and Table 8 present the data generated in a successful round of experiment Phase 2.

Unlike Phase 3, in Phase 2, the auction happened periodically, generating more data. This becomes evident by comparing the data generation in round 2 of Phase 3 (Table 5 and Table 6) with the **successful** round of Phase 2 (Table 7 and Table 8). Even with fewer network participants, the round in Phase 2 generated just 26 MB less in the orderer and 76 MB more in the peer.

Therefore, to achieve better precision, we utilize the Phase 2 data generation and execution time to make estimates for longer periods. As stated in Listing 3, each sensor published **20** transactions plus one declaring itself active. Each seller performed **5** energy generation and **5** sell bid publication transactions, while the buyers submitted **10** buy bids and **10** buy bid validation transactions. **Twenty-nine** auctions were executed in the successful Phase 2 round.

Considering that the round took 27 min to complete and generate the data quantity presented in Table 7 and Table 8, we could estimate how much storage would be required to support the network execution with the same configurations for longer periods. The estimates for the data generated in a day, month, and year are presented in Table 9. Such estimates were calculated linearly, as the applications and the chaincode generate data linearly.

The estimated transaction numbers are also presented in the Table 9 rightmost column, excluding the first row, since it consists of a measure and not an estimate. Even though the Ethereum blockchain has a pretty different concept, it serves as a suitable anchor for comparing data generation and transaction quantity. In the first half of 2021, the Ethereum main network grew by approximately 1 Gigabyte (GB) per day, with around 1.2 million transactions and the rate of 1 GB per million transactions.

Meanwhile, our network generated data at 8 GB per million transactions. With only a single endorser signing the transactions in our experiments, this rate could increase proportionally to the endorsers’ quantity in other scenarios. Solutions to deal with such characteristics would enhance the proposed model and increase its adoption chance.

We consider the idea of multi-layer chains (or channels) as a possible solution. The upper-level chains could perform some digest on the lower chains’ transactions and store it. The raw data could last for a specific time interval and, after that, be digested, referenced in the upper chain, and erased from the lower chain.

In our experiments’ context, the lower chain is equivalent to the network proposed and implemented by this work. The upper chain could be developed by future work. This architecture fits well with an energy trading scenario that does not require high granularity for long periods. As a result, the data generation rate could be lowered.

### 7.4. Energy Network Baseline Cost Analysis

Evaluating the costs of a proposal is crucial for judging its feasibility. For that reason, we estimate the cost of deploying our model. Based on the *c6g.8xlarge* AWS instances costs, we estimate the funds needed to run a network equivalent to the 4th round of Phase 3 in terms of execution cost. Nevertheless, since we focused our data generation analysis on the Phase 2 round, it will serve as a reference to estimate the storage costs.

The *c6g.8xlarge* instance *on-demand* costs of 0.6816 USD hourly. Regarding storage, we consider the pricing for an AWS General Purpose SSD (gp2) Elastic Block Store (EBS), which is 0.114 USD per GB-Month. All costs are related to the Mumbai region and are displayed in Table 10. In the first three rows, we assumed that the storage space was **fully** provisioned initially. However, we considered storage increases on a month-by-month demand basis for the *year* cost row. Equation (1) expresses the formula to calculate it as a 12-term sum of the monthly cost arithmetic progression.

(1) $$Ysc=\frac{(GBMcxMgx12+GBMcxMg)*12}{2}$$

Based on the yearly total cost and transaction rates, our model with one peer and one orderer costs (USD) per transaction ratio of $9.92\times 10^{-6}$. Considering an average Ethereum transaction fee of 5 USD while disregarding the 2021 transaction fee volatility [32], our network presents significantly lower costs. The comparison holds even if compared against Ethereum’s lowest historical transaction fee of 0.5 USD.

Unlike our experiments, a real Hyperledger Fabric network would have more than one peer and one orderer. Presuming that the real network would have 20 peers and 20 orderers to process the same 2.8 B, the cost per transaction would be around the value of $1.94\times 10^{-4}$ USD, which still surpasses Ethereum (this is a rough estimate without considering that the transaction size and performance would be affected with more peers and orderers in the network).

If the solution mentioned in Section 7.3 about reducing the data generation rate is implemented, the costs could drop. Furthermore, other storage solutions like AWS S3 or EFS should be analyzed in our model’s context. The EBS has a maximum capacity of 16 Tebibyte (TiB) per volume, and changing the storage tools would change network prices.

### 7.5. Energy Network Viability

We achieved a successful throughput of 5000 sellers, 5000 sensors, and 5000 buyers simultaneously. At these metrics, our proposed model suits a **small neighborhood**. The proportion between sensors and buyers/sellers in our experiments may differ in a real environment since many more buyers/sellers are expected than sensors.

The energy validation transactions consume a considerable amount of chaincode processing. For that reason, blockchain trading models without energy validation based on sensors’ data might attain greater performance. However, this decision depends on the network architect’s objectives of applying blockchain in the energy context.

We assumed that validating energy before the sale would prevent fraud and increase the trust in the energy generation type, serving as a helpful feature. Buyers’ anonymization might be a regulator’s requirement to protect users according to data privacy laws, and our implementation covers it.

Our analysis intends to provide **computational** and **cost** perspectives of blockchain use in energy trading. Energy engineering researchers might consider our findings and judge if blockchain fits this area due to their greater knowledge in the field.

### 7.6. Security Achievements

Using blockchain in energy markets already brings some intrinsic security properties. Among these properties are the availability and non-repudiation due to the distributed ledger and public-key cryptography on transactions. However, our model also has its own security achievements, plus security characteristics related to Hyperledger Fabric.

Fabric does not support the Byzantine consensus. Thus, our model is susceptible to an attack if there is any malicious node. Nevertheless, we assume that the entities controlling nodes in the network, like energy companies, know one another and have a considerable degree of trust. They are incentivized to act accordingly in this environment, or otherwise litigation may occur. An alternative to mitigate the Byzantine consensus absence could be performing crosschaining by periodically storing the block hashes on a public chain.

With x509 certificate attributes, our model’s chaincode implements access control, allowing only entities with proper attributes to call specific functions. This characteristic prevents, for example, an energy seller from calling chaincode functions designated to sensors, trying to tamper with the network. The responsibility for maintaining the attributes correct in the certificates falls on each organization’s CA.

The main security achievements are energy validation and buyers’ anonymization. Whenever sellers desire to validate the generated energy, they must collect sufficient endorsements from multiple organizations validating that claim. The endorsements’ quantity may vary according to the network’s policy. We assume that if several organizations query their trusted sensors and conclude that the energy is valid, the network can trust that it originated from a clean source. This validation scheme prevents sellers from connecting, for example, a diesel generator on a cloudy day and trying to sell the same amount of solar energy as on a sunny day.

The energy consumption patterns exposure is solved in our model by anonymizing the buyers. With Idemix, two consecutive buyers’ transactions cannot be linked, avoiding consumption pattern exposure. Still, buyers can prove the ownership of their credentials and break their anonymity if they desire. In our model, this is important for a buyer to prove to the utility company that they acquired clean energy.

Overall, our model enables energy traceability. All energy generated is linked to a location, a seller, a source type, the organizations that validated it, and what sensors were consulted. This information increases the trust in the clean energy generation process and might be required by regulators or consumers. Furthermore, how the information is validated in our model might apply to other problem domains.

### 7.7. Related Work Comparison

The proposals for related work are heterogeneous, with different market designs, experiment complexity, and focus. However, this work contributed to the research done by their authors in many diverse aspects like privacy, scalability, experiment depth, experiment procedure, and empirical data. We compare our work’s implementation and results with the related work based on their proposals, experiments, and future directions. Table 11 presents the comparison described in the this subsection and its symbols must be interpreted like Table 1.

The authors of [11,14,17] only proposed or implemented simple models regarding blockchain in energy markets, and we brought clarity to a topic that lacks experimental data. As suggested by [14], our work did not use PoW consensus. Even though our model was implemented with a single chain, different from [11,13], we consider a multi-chain approach for dealing with the data quantity due to our model’s data generation rate.

We implemented a solution with pseudonymity, as suggested by [24] future directions, and with off-chain payment to enable the pseudonymity, guaranteeing the funds, as mentioned by [13]. We did not implement a day-ahead and real-time market similar to [18] because we focused on validating the energy before exchanging.

The most interesting comparison is with the thesis of [22]. They implemented an energy market with an Ethereum smart contract using PoW consensus, which consumes more power than the alternatives and should be avoided in a clean energy context. Furthermore, the authors implemented a model with off-chain market clearing. With these characteristics, they simulated their implementation with 600 entities transacting simultaneously.

Our implementation keeps the market clearing in the chain and adds the energy validation process based on sensors’ data. Despite these smart contract processing increases, we could handle 15,000 entities transacting simultaneously. Unlike [22]—with day-ahead, real-time, and load curtailment markets—our model only lets energy generated in the past be exchanged. Therefore, our exchange options implied a lower chaincode complexity in this aspect, perhaps helping with the higher throughput.

In terms of cost, the [22] required 8 billion Ethereum gas for a network with 600 entities and a 15-min market clearing. Considering a gas price of 15.8 Gwei and an Ether price of 2031 USD, their proposal would cost around 250,000 USD per day if it ran in the Ethereum main net. Meanwhile, if the yearly costs of our experiment are divided by the days in a year, our model costs can be estimated to be 76 USD per day for each pair of peers and orderers.

By accomplishing the [22] future directions, our model could achieve higher scalability and better privacy. It was tested with real computers, even though we did not use real smart meters. The data in Table 10 points to the best throughput of 93 transactions per second by our implementation. A throughput of 6 transactions per minute was seen in [16], while [22] mentions the need for 52 transactions per second throughput, both in an Ethereum energy trading scenario.

While [21] focused on the IoT component of blockchain energy trading, we did not take our model and experiments that far. Future work could research lighter interactions between restricted IoT devices and blockchain networks. The Hyperledger communication stack, including the Fabric SDKs, seems too heavy for lightweight devices.

## 8. Conclusions

### 8.1. Conclusions and Contributions

In this work, we proposed, implemented, and analyzed a blockchain-based energy trading scheme with validation using IoT sensors data. The negotiated energy must have been generated in the past and has a significant guarantee of origin. This is accomplished by a decentralized multi-organizational chaincode which requires a minimum organization quorum to validate energy generation claims.

In our implementation, to protect buyers’ energy consumption patterns, they transact with the network through a k-TAA algorithm (idemix). Even though the energy cannot be bought on-demand in our model, the buyer anonymization facilitates the implementation of a future secure real-time blockchain energy market.

We analyzed our solution’s performance, scalability, and costs, considering different quantities of sensors, buyers, sellers, and different hardware configurations for peers and orderers. Some Hyperledger configurations like the peer concurrency limit, the memory allocated for the chaincode, block size, and block interval also were changed and analyzed. If we consider our results, our solution fits a small neighborhood context.

Our model’s better throughput and estimated cost indicate that a solution with Hyperledger Fabric is more efficient than the Ethereum solutions presented by the related work. With a single peer and a single orderer, we measured a cost per transaction outstandingly lower than the one charged by the Ethereum main net.

As secondary contributions, we developed scripts that easily deploy configurable Hyperledger networks, enabling parameters like organizations, peer quantity, orderer quantity, and chaincodes to be defined. The certificates host fields are set according to the hosts attributed by the cloud service. These scripts contribute to future work that depends on deploying a Hyperledger network on a cloud infrastructure similar to AWS.

Our Fabric modifications contribute to previous and future research. We enabled idemix in the fabric-gateway-java by implementing the required interfaces and performing minor alterations on the fabric-sdk-java. The transactions, with our modifications, have priorities that are set by the chaincode function return.

It is now possible to bypass the *phantom read* checks by setting the proper chaincode function return method. Future work with Hyperledger chaincodes might take advantage of this modification to avoid time-costly transactions that are wrongfully invalidated due to the *phantom read conflicts*. Thus, the support for more complex chaincodes is increased.

Finally, we analyzed the impact of the Hyperledger Fabric database type choice. With our chaincode, Go LevelDB presented a significantly better performance than CouchDB. While CouchDB supports enhanced queries, it is quicker to retrieve data and implement the sorting in the chaincode using LevelDB. However, the more limited key queries with LevelDB require the proper design of data keys, or it might not be easy to perform attribute-based queries.

### 8.2. Future Work

Since knowing the precise function to calculate the maximum possible energy generated by a specific solar panel type, or wind turbine, was out of our scope, we leave it as future work for researchers in electrical engineering. The network consensus on how much energy a specific solar panel model can generate given environment metrics could also be designed and implemented.

Hyperledger Fabric allows the change of multiple configuration parameters, and we did not explore their full extent. Further analysis on enhancing performance through better Fabric configuration would add more reliability to our work. Also, there is space for experiments with more organizations, peers, orderers, sensors, and buyers, which might require model modifications to handle higher transaction throughput.

In our experiments, the utility and payment companies’ HTTP servers were removed to increase the reliability of the blockchain performance metrics. To fully validate our solution, new experiments, including the HTTP servers, would be required. However, some challenges come with bringing them back.

Golang HTTP servers would fit much better in terms of scalability and concurrent request handling. Still, at the moment, only fabric-java-sdk provides idemix support, and the utility company server performs idemix signature verifications. Idemix support for the fabric-sdk-go would facilitate setting scalable HTTP servers.

All sensors are retrieved from the database as possible participants of an energy validation claim process in our implementation. Then the chaincode calculates the sensor distance to the seller and judges if the sensor will participate or not. Instead, a geospatial database could take the seller’s location as query input and only return the near sensors more efficiently, as we suppose.

The SmartData provides the *confidence* and *error* fields, but we do not evaluate them in our chaincode. Future work could consider these fields and give more weight to SmartData with bigger confidence and discard the SmartData with error. Such verification would increase the energy validation reliability.

Furthermore, SmartData version 1.2 supports data from moving sensors. In the current implementation, our chaincode considers all sensors as static data sources. However, extending it to moving ones could enhance the energy validation process, but would also require more analysis.

The energy sold through our chaincode has to be generated in the past. Nevertheless, the following work could use our findings as a base to experiment with a futures energy market with energy delivery verification. This would bring blockchains closer to the current energy markets.

In a hypothetical energy futures market, like the day-ahead, parties would agree on an energy amount for a specific price in some future time. The market designed by us could serve as a way to clear settlements. For example, if sellers realize that they are incapable of delivering the promised energy amount, they could buy energy in our proposed present market to settle the agreement.

Our previous work [33] analyzed CoAP and DTLS on an IoT gateway, using UDP, considered a more efficient protocol for constrained devices. Meanwhile, Hyperledger Fabric communicates through gRPC, which uses TCP. An examination of sensor gateways running gRPC would be relevant. Perhaps, a solution considering IoT-friendly protocols could enhance the interaction between gateways and blockchains.

In the chaincode, sellers, sensors, and companies are uniquely identified by the Base64 encoding of the certificate Distinguished Names, which usually generates a string sized around 176 bytes. A smaller unique identification would promote more efficient stores in Fabric’s database, considering that, for example, every SmartData record stores the sensor’s identification string.

We could not find any energy consumption per instance type in AWS EC2 documentation. The energy spent on our models’ execution would be an interesting metric to decide if our model is efficient from an energetic and environmental standpoint. The clean energy amount negotiated and incentivized by the blockchain market should be worth the energy spent executing the market’s infrastructure.

The authors of [34] present some adaptations of Hyperledger Fabric source code that scale up its throughput to a rate of 20,000 transactions per second. Since some of our chaincode’s transactions require considerably more processing than regular Hyperledger Fabric transactions, future work could run our experiments with [34] adaptations to verify if the throughput would increase. 

## Figures and Tables

**Table 1:** Related work comparison.

	Characteristics	Permissioned	Deals with Privacy	Deals with Scalability	Implemented	Ethereum	Hyperledger	Depth of Market Design
	Related Work							
[11]		✗	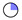			✗		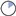
[12]		✗	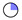		✗	✗		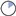
[13]		✗	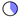	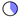				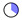
[14]		✗	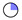			✗		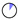
[15]				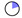				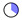
[16]		✗	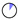	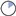	✗	✗		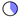
[17]		✗	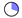	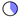			✗	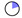
[18]		✗	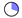	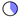	✗		✗	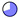
[20]		✗	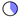	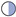		✗		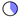
[21]			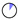	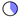		✗		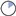
[22]		✗	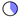	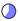	✗	✗	✗	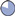
[23]			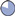					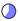
[24]		✗	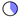	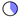		✗		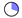
This work		✗	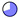	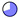	✗		✗	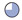

**Table 2:** Configurations that lead to failure in Phase 1. The instances which indicated failure are highlighted in red.

	Settings	Round Number	App Instance	Orderer Instance	Peer Instance	Number of Sellers	Number of Sensors	Number of Buyers
	Who Failed							
application		1	t4g.micro	t4g.micro	t4g.micro	200	200	200
peer		2	t4g.xlarge	t4g.micro	t4g.micro	400	400	400
orderer		3	t4g.xlarge	t4g.micro	t4g.xlarge	600	600	600
peer		4	t4g.xlarge	t4g.xlarge	t4g.xlarge	600	600	600
application		5	t4g.xlarge	t4g.xlarge	t4g.2xlarge	600	600	600
orderer		6	t4g.2xlarge	t4g.xlarge	t4g.2xlarge	800	800	800
peer (concurrency limit)		7	t4g.xlarge	t4g.2xlarge	t4g.2xlarge	1000	1000	1000

**Table 3:** Configurations that lead to failure in Phase 2. The instances which indicated failure are highlighted in red.

	Settings	Round Number	App Instance	Orderer Instance	Peer Instance	Number of Sellers	Number of Sensors	Number of Buyers
	Who Failed							
application		1	t4g.2xlarge	t4g.2xlarge	t4g.2xlarge	2000	2000	2000
orderer		2	c6g.4xlarge	t4g.2xlarge	t4g.2xlarge	2000	2000	2000
peer		3	c6g.4xlarge	c6g.4xlarge	t4g.2xlarge	2700	2700	2700
peer (chaincode mem limit 2 GiB)		4	c6g.4xlarge	c6g.4xlarge	c6g.4xlarge	3000	3000	3000
application/peer		5	c6g.4xlarge	c6g.4xlarge	c6g.4xlarge	3500	3500	3500
application		6	c6g.8xlarge	c6g.4xlarge	c6g.8xlarge	3500	3500	3500
orderer		7	c6g.16xlarge	c6g.4xlarge	c6g.8xlarge	3500	3500	3500

**Table 4:** Round configurations in Phase 3. The instances which indicated failure are highlighted in red, while green highlight the successful rounds.

	Settings	Round Number	App Instance	Orderer Instance	Peer Instance	Number of Sellers	Number of Sensors	Number of Buyers	Block Interval	Block Transaction Limit	Block Preferred Size
	Who Failed										
application (add new app instance)		1	c6g.16xlarge	c6g.8xlarge	c6g.8xlarge	3500	3500	3500	10 s	2000	60 MB
success		2	c6g.16xlarge	c6g.8xlarge	c6g.8xlarge	3500	3500	3500	10 s	2000	60 MB
orderer (Low block transaction limit)		3	c6g.16xlarge	c6g.8xlarge	c6g.8xlarge	5000	5000	5000	10 s	2000	60 MB
success		4	c6g.16xlarge	c6g.8xlarge	c6g.8xlarge	**5000**	**5000**	**5000**	10 s	10,000	60 MB
peer (chaincode mem limit 8 GiB)		5	c6g.16xlarge	c6g.8xlarge	c6g.8xlarge	6000	6000	6000	10 s	10,000	60 MB
orderer		6	c6g.16xlarge	c6g.8xlarge	c6g.8xlarge	6000	6000	6000	10 s	10,000	60 MB
orderer		7	c6g.16xlarge	c6g.16xlarge	c6g.8xlarge	6000	6000	6000	10 s	20,000	90 MB
orderer		8	c6g.16xlarge	c6g.16xlarge	c6g.8xlarge	6000	6000	6000	20 s	30,000	150 MB

**Table 5:** Orderer data generation in Phase 3.

	Settings	Number of Sellers	Number of Sensors	Number of Buyers	Initial Size	Final Size	Data Generated
	Phase 3 Round						
2 (success)		3500	3500	3500	289 MB	897 MB	608 MB
4 (success)		5000	5000	5000	289 MB	1178 MB	889 MB

**Table 6:** Peer data generation in Phase 3.

	Settings	Number of Sellers	Number of Sensors	Number of Buyers	Initial Size	Final Size	Data Generated
	Phase 3 Round						
2 (success)		3500	3500	3500	53 MB	597 MB	544 MB
4 (success)		5000	5000	5000	53 MB	833 MB	780 MB

**Table 7:** Orderer data generation in successful Phase 2 round.

	Settings	Number of Sellers	Number of Sensors	Number of Buyers	Initial Size	Final Size	Data Generated
	Phase 2 Round						
success		3000	3000	3000	1564 MB	2146 MB	582 MB

**Table 8:** Peer data generation in successful Phase 2 round.

	Settings	Number of Sellers	Number of Sensors	Number of Buyers	Initial Size	Final Size	Data Generated
	Phase 2 Round						
success		3000	3000	3000	1318 MB	1928 MB	610 MB

**Table 9:** Data generation and transaction estimates based on the successful Phase 2 round.

Generation Period	Orderer Generated	Peer Generated	Orderer + Peer	Transactions
27 min	582 MB	610 MB	1192 MB	150 K
1 day	31 GB	32.5 GB	63.5 GB	8 M
1 month	930 GB	975 GB	1.8 TB	240 M
1 year	11 TB	11.7 TB	22.7 TB	2.8 B

**Table 10:** Cost estimate based on round 4 of Phase 3 execution, but Phase 2 data generation and transaction rate.

Execution Period	Orderer/Peer Single Instance Cost (USD)	Instances Total Cost (USD)	EBS Cost for Generated Data USD	Total Cost (USD)	Transactions
27 min	0.3	0.6	0.114	0.714	150 K
1 day	16.35	32.70	7.24	39.94	8 M
1 month	490.5	981	205.2	1186.2	240 M
1 year	5886	11,772	16,005	27,777	2.8 B

**Table 11:** Results comparison with related work.

	Model Elaboration	Consensus	Multi-Chain	Pseudonymity	Throughput (tps)	Dayli Cost (USD)	Day-Ahead
[11]	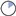	PoW	✗				
[13]	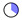		✗				
[14]	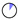	Not PoW					
[16]	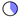	PoW			6		
[17]	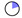	Not PoW					
[18]	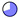	Not PoW					✗
[21]	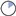						
[22]	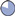	PoW			52	250 K	✗
[24]	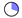	-	-	✗			
This work	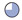	Not PoW	No	✗	93	76 *	No **

* per orderer and peer pair; ** Our market can be used to clear a hypothetical futures (day-ahead) market.

## Data Availability

The data that support the findings of this study are available on request from authors.

## Abbreviations

The following abbreviations are used in this manuscript:

AMI	Amazon Machine Image
API	Application Programming Interface
AWS	Amazon Web Services
BRP	Balance Responsible Party
CA	Certificate Authority
CLI	Command-Line Interface
CoAP	Constrained Application Protocol
CPU	Central Process Unit
DLL	Dynamic-link Library
DNS	Domain Name System
DRBG	Deterministic Random Bit Generator
DSO	Distribution system operator
DTLS	Datagram Transport Layer Security
EBS	Elastic Block Store
EC2	Elastic Compute Cloud
GB	Gigabyte
GBMc	AWS charge for monthly gigabyte storage allocation (USD)
Gbps	Gigabits per second
GiB	Gibibyte
gRPC	Remote Procedure Calls
HTTP	Hypertext Transfer Protocol
IoT	Internet of Things
IP	Internet Protocol
JSON	JavaScript Object Notation
KB	Kilobyte
k-TAA	k-Times Anonymous Authentication
kWh	Kilowatt-hour
MPC	Multiparty Computation
MSP	Membership Service Provider
OU	Organizational Unit
P2P	Peer-to-Peer
PoA	Proof-of-Authority
PoS	Proof-of-Stake
PoW	Proof-of-Work
protobuf	Protocol Buffers
RAM	Random Access Memories
SDK	Software Development Kit
SI	International System
SSD	Solid-State Drive
SSH	Secure Shell
TB	Terabyte
TCP	Transmission Control Protocol
TiB	Tebibyte
TLS	Transport Layer Security
tps	Transactions per second
TSO	Transmission system operator
UDP	User Datagram Protocol
USD	United States dollars
YAML	YAML Ain’t Markup Language
Yg	Monthly data generation (GB)
Ysc	Yearly storage cost (USD)
